# Increased CCL19 and CCL21 levels promote fibroblast ossification in ankylosing spondylitis hip ligament tissue

**DOI:** 10.1186/1471-2474-15-316

**Published:** 2014-09-26

**Authors:** Yang Qin, Li Da He, Zhou Jian Sheng, Miao Ming Yong, Yang Sheng Sheng, Xu Wei Dong, Tong Wen Wen, Zou Yu Ming

**Affiliations:** Department of Orthopedics, Changhai Hospital Affiliated to the Second Military Medical University, Changhai Road 168, Shanghai, 200433 Yangpu district P.R. China; School of Kinesiology, Shanghai University of Sport, Hengren Road 200, Shanghai, 200438 Yangpu District P.R. China; Department of Biochemistry and Molecular Biology, Second Military Medical University, Xiangyin Road 800, Shanghai, 200433 Yangpu District P.R. China

**Keywords:** CCL19, CCL21, Ankylosing spondylitis, Fibroblast, Ossification

## Abstract

**Background:**

It is well-documented that both chemokine (C-C motif) ligand 19 (CCL19) and 21 (CCL21) mediate cell migration and angiogenesis in many diseases. However, these ligands’ precise pathological role in ankylosing spondylitis (AS) has not been elucidated. The objective of this study was to examine the expression of CCL19 and CCL21 (CCL19/CCL21) in AS hip ligament tissue (LT) and determine their pathological functions.

**Methods:**

The expression levels of CCL19, CCL21 and their receptor CCR7 in AS (n = 31) and osteoarthritis (OA, n = 21) LT were analyzed via real-time polymerase chain reaction (RT-PCR) and immunohistochemistry (IHC). The expression of CCL19, CCL21 and CCR7 in AS ligament fibroblasts was also detected. The proliferation of ligament fibroblasts was measured via a cell counting kit-8 (CCK8) assay after exogenous CCL19/CCL21 treatment. Additionally, the role of CCL19/CCL21 in osteogenesis was evaluated via RT-PCR and enzyme-linked immunosorbent assay (ELISA) in individual AS fibroblast cultures. Furthermore, the expression of the bone markers alkaline phosphatase (ALP), osteocalcin (OCN), collagenase I (COL1), integrin-binding sialoprotein (IBSP) and the key regulators runt-related transcription factor-2 (Runx-2) and osterix were investigated. Moreover, the CCL19/CCL21 levels in serum and LT were measured via ELISA.

**Results:**

The mRNA levels of CCL19/CCL21 in AS hip LT were significantly higher than that in OA LT, and IHC analysis revealed a similar result. Exogenous CCL19/CCL21 treatment did not affect the proliferation of ligament fibroblasts but significantly up-regulated the expression of bone markers, including ALP and OCN, and the key regulators Runx-2 and osterix. In addition, the serum levels of CCL19/CCL21 were apparently elevated in AS patients compared to healthy controls (HC), and the expression of the two chemokines correlated significantly in AS patients.

**Conclusions:**

CCL19 and CCL21, two chemokines displaying significantly associated expression in serum, indicating a synergistic effect on AS pathogenesis, may function as promoters of ligament ossification in AS patients.

**Electronic supplementary material:**

The online version of this article (doi:10.1186/1471-2474-15-316) contains supplementary material, which is available to authorized users.

## Background

Ankylosing spondylitis (AS) is the representative rheumatic disease of seronegative spondyloarthropathy and is characterized primarily by recurrent inflammatory back pain and bilateral sacroiliitis [1]. Hip ankylosis, which is typically accompanied by enthesis heterotopic ossification (HO), occurs in about one-third of patients suffering from AS [2]. Entheopathy in peripheral and axial sites induces distinct pathologic changes, and inflammatory enthesitis is clinically detectable in approximately 10% of patients suffering from early-stage AS and 50% of those suffering from established AS [3]. Fibroblasts are the most numerous connective tissue cells in enthesis or ligament tissue (LT) and are reported to be associated with heterotopic ossification (HO) in LT [4, 5]. However, the relevant mechanism by which fibroblasts mediate HO remains unclear.

CCL19, CCL21 and their corresponding receptor CCR7 are described to be crucially involved in the adaptive immune system, their primary role being the migration of monocytes/macrophages, mature dendritic cells (DCs) and naive T cells to lymph nodes [6–9]. Studies have demonstrated that CCR7 is responsible for the migration of T cells into inflamed tissues and T-cell egress from these tissues via the afferent lymph under inflammatory conditions [10] and that CCR7 knockout attenuates the development of diseases such as coronary sclerosis [11]. Moreover, CCR7 signaling is considered to mediate both angiogenesis and tumor metastasis in different tumor microenvironments [12–14].

The effects of these two chemokines on proliferation, adhesin and/or integrin avidity, invasiveness, endocytosis, differentiation and survival have been comprehensively summarized in a prospective review [15].

Numerous studies have revealed their respective roles in immune diseases, such as rheumatoid arthritis (RA), eosinophilic pneumonia, breast cancer and acquired immune deficiency syndrome (AIDS) [16–18]. A recent study demonstrated that CCL19 was more strongly expressed in systemic sclerosis (SSc) skin and was correlated to vascular inflammation [19], providing further evidence for the role of CCL19 in perivascular inflammation and immune cell recruitment. A parallel study illustrated that serum CCL19 levels might reflect blood B cell imbalance and correlate to the levels of some serum B cell biomarkers, such as rheumatoid factor, anti-CCP, free light chains, IgG, IgA and IgM, in RA patients. Furthermore, serum CCL19 also acted as a potential predictor of the clinical response to rituximab in B cell-mediated RA subtypes [20]. All of these results illustrate the pivotal function of CCL19 in clinical settings. However, the role of CCL19/CCL21 in ankylosing spondylitis is rarely reported and demands a more comprehensive understanding.

In this study, we preliminarily focus on AS patients experiencing severely limited hip activity (average BASFI = 62.24, which means severe functional limitations). First, we detected higher expression of CCL19, CCL21 in LT and ligament fibroblasts. No significant effect on LT fibroblast proliferation was detected upon exposure to these two chemokines. Next, we examined the roles of CCL19/CCL21 in the osteogenic potential of AS ligament fibroblasts. The expression of the bone markers ALP, OCN, Runx-2 and Osterix were up-regulated by CCL19/21 simulation. However, IBSP and COL1 expression was not seen up-regulated. Serum CCL19/CCL21 levels are closely associated, and CCL19 expression appeared to correlate to the Visual Analogue Scale (VAS) Pain Score and disease duration, whereas CCL21 expression did not display this correlation. Our result reveals a novel role of CCL19/CCL21 in ligament ossification and may provide useful evidence for further investigation in AS patients.

## Methods

### Patients

Patients meeting the modified New York criteria [21] for AS and patients suffering from single hip OA who underwent total hip arthroplasty at our department from May 1, 2012, to October 31, 2013, were selected for this study. The demographic and clinical characteristics of all the subjects are summarized in Table 1. All patients who provided their written informed consent according to the Declaration of Shanghai Changhai Hospital were enrolled. This study was approved by Shanghai Changhai Hospital Ethics Committee and the Ethics Committee approval document number is CHEC2013-194 The LT samples were stored in a −80°C freezer after collection in the operating room. Primary cultures of ligament fibroblasts were generated according to a previously described method [22] with some modifications.

**Table 1:** **Demographic characteristics of AS and OA patients and healthy controls**

	Ankylosing spondylitis (AS) (n = 44)	Osteoarthritis (OA) (n = 29)	Healthy controls (HC) (n = 16)
Male/female	35/7	20/9	9/7
Age (y)	35.93 ± 11.30	43.48 ± 11.21	33.38 ± 5.88
HLA-B27 (+)	36	NA	NA
ESR (mm/h)	42.29 ± 25.74	8.92 ± 8.26	NA
CRP (mg/L)	25.41 ± 19.75	9.56 ± 21.96	NA
BASDAI score	52.3 ± 14.7	NA	NA
BASFI score	62.24 ± 17.98	NA	NA
VAS score	5.26 ± 1.94	NA	NA
BAS-G score	5.51 ± 1.56	NA	NA
Disease duration (months)	155 ± 103	NA	NA
Morning stiffness (min)	13 ± 16	NA	NA
Serum CCL19 (pg/ml)	354.12 ± 75.49	304.55 ± 82.82	215.15 ± 50.20
Serum CCL21 (pg/ml)	535.33 ± 124.22	470.13 ± 79.75	390.92 ± 38.12

The data are expressed as the means ± Standard deviation (SD). *ESR*: Erythrocyte sedimentation rate; *CRP*: C-reactive protein; *BASDAI*: Bath Ankylosing Spondylitis Disease Activity Index; *BASFI*: Bath Ankylosing Spondylitis Functional Index; *VAS*: Visual analog scale; *BAS-G*: Bath Ankylosing Spondylitis Patient Global Score. *NA*: Not applicable.

### RNA extraction and real-time PCR

The LT was fully shredded using RNase-free scissors and transferred to a 1.5 ml RNase-free EP tube. A total of 1 ml of TRIzol (Invitrogen) was used for no more than 100 mg of LT. TRIzol was used according to the manufacturer’s instructions. Total RNA was reverse-transcribed to cDNA using a ReverTra Ace qPCR RT Kit (TOYOBO CO., LTD., Osaka, Japan). The transcripts were stored at −80°C until further use. For RT-PCR, SYBR Green Real Time PCR Master Mix (TOYOBO CO., LTD., Osaka, Japan) was used in a total volume of 20 μL. The primer sequences are listed in Table 2. The samples were evaluated in triplicate using an equal load of 10 ng of cDNA/well, and three independent experiments were performed. The data were collected using an ABI 7300 Real-Time PCR System.

**Table 2:** **Primer sequences used for real-time PCR**

	Sense primer (5′ to 3′)	Anti-sense primer (5′ to 3′)
Human CCL19	CCAACTCTGAGTGGCACCAA	TGAACACTACAGCAGGCACC
Human CCL21	TGGCCTCTTACTCACCCTCT	GCCTCTTGATCCCCTTAGCC
Human CCR7	GCCTACGACGTCACCTACAG	GGCAGAAGAGTCGCCTATGG
Beta-actin	CCATCGTCCACCGCAAAT	TGTCACCTTCACCGTTCC
ALP	ATCTTTGGTCTGGCTCCCATG	TTTCCCGTTCACCGTCCAC
Human OCN	GGCAGCGAGGTAGTGAAGAG	CTGGAGAGGAGCAGAACTGG
Human COL1	GAG AGC ATG ACC GAT GG	GTG ACG CTG TAG GTG AA
IBSP	CGCCAATGAATACGACAATG	GATGCAAAGCCAGAATGGAT
Human VEGF-A	GAGCCTTGCCTTGCTGCTCTA	CACCAGGGTCTCGATTGGAT
Runx-2	GCAGCAACCCAGAAACACTT	AACACATGACCCAGTGCAAA
Osterix	AGAGGAGAGACTCGGGACAG	GAGTTGTTGAGTCCCGCAGA

*CCL19*: Chemokine (C-C motif) ligand 19; *CCR7*: Chemokine receptor 7; *ALP*: Alkaline phosphatase; *OCN*: Osteocalcin; *COL1*: Collagenase I; *IBSP*: Integrin-binding sialoprotein; *VEGF-A*: Vascular endothelial growth factor-A; *Runx-2*: Runt-related transcription factor-2.

### Immunohistochemistry

The LT samples were immediately fixed using 4% formaldehyde and embedded in paraffin. The samples were deparaffinized in xylene for 20 minutes at room temperature, followed by rehydration using an alcohol gradient. Antigens were unmasked by first incubating the slides in 0.01 mol/L boiling citrate buffer at pH 6.0 for 15 minutes and then cooling at room temperature. Endogenous peroxidase activity was blocked via incubation in 3% H_2_O_2_ for 5 minutes, followed by blocking with goat serum for 30 minutes at 37°C. The primary antibody rabbit anti-human CCL19 (1:100, R&D Systems), rabbit anti-CCL21 (1:100, R&D Systems), rabbit anti-CCR7 (1:100, ABGENT), or an isotype-matched irrelevant antibody (R&D Systems) was individually added to the sections at the proper concentration. Following three washes with PBS, the slides were incubated for 30 min in the secondary antibody (GT Vision III anti-mouse/rabbit Universal immunohistochemical detection kit, Dako, Denmark) at 37°C. The slides were washed 3 times with PBS and then stained with DAB for 5 minutes and hematoxylin for 45 s, followed by rinsing with tap water for 5 minutes. Finally, the slides were processed via alcohol gradient dehydration, xylene immersion and resin blocking. Three fields from each slide were randomly selected, and tissue staining was analyzed using Image-Pro Plus 6.0 software. The scored data were pooled, and the mean density value ± standard deviation (SD) was calculated for each group.

### Primary cell isolation, culture, and procedures

The previously described method for primary cell isolation was mentioned performed [22] with some modifications. The LT from patients suffering from AS or OA who underwent hip joint replacement surgery was washed with sterile saline to remove the blood. Then, the fats and synovial tissue were cleared from the LT, followed by shredding into 1–2 mm^3^ tissue blocks. The tissue blocks were centrifuged twice in 15 ml centrifuge tubes in 10 ml of PBS at 1000 r/min. Next, the tissue blocks were digested in 5 ml of PBS and 500 μl of collagenase I (Sigma-Aldrich China, Shanghai, China) for 5 h at 37°C. Then, 5 ml of 0.25% trypsin was added, and the samples were mixed and incubated at 37°C for 2 minutes. The samples were filtered using 200 mesh filters, and the filtrate was centrifuged at 1000r/m for 5 minutes. The supernatant was discarded. Then, 5 ml of DMEM/high glucose culture medium supplemented with 100 U/ml penicillin (Invitrogen, Carlsbad, California, USA), 100 μ g/ml streptomycin (Invitrogen, Carlsbad, California, USA) and 12% FBS was added to the centrifuge tube. The samples were mixed by pipetting and transferred to culture flasks in a humidified 5% CO2 incubator at 37°C The passages from 3 to 8 were used. For osteogenic differentiation, the fibroblasts were cultured to confluence and then incubated in DMEM/high glucose medium containing 100 nM dexamethasone, 50 μg/ml ascorbic acid, 10 mM of β-glycerophosphate [23, 24] and 12% fetal bovine serum.

### Flow cytometric analysis

The expression of CD90 (Biolegend) was identified as a molecular marker of fibroblasts based on FACS analysis as previously described [25, 26]. The fibroblasts were detached via brief incubation in 2 mM EDTA in PBS. Following fixation using 1 ml of fix buffer (0.5% BSA-PBS) and incubation at room temperature for 10 min, approximately 1 × 10^6^ cells were stained at room temperature via incubation in an anti-CCR7 pAb (polyclonal antibody, catalog # AP4998a, ABGENT) or an irrelevant IgG2a isotype control at a concentration of 0.025/ml for 90 min. After two washing and centrifugation steps, the cells were incubated in a PE-conjugated goat-anti-rabbit IgG pab (Tianjin Sungene Biotech Co., Ltd, Tianjin, China) for 40 min. Then, the cells were immediately analyzed via flow cytometry, and the data were calculated using a FACSCalibur flow cytometer and CellQuest software (BD Biosciences).

### Measurement of fibroblast proliferation

The proliferation of fibroblasts was examined via a cell counting kit-8 (CCK-8 kit) assay according to the manufacturer’s instructions. Briefly, approximately 2 × 10^3^ cells were seeded in a volume of 100 μl DMEM on each well of a 96-well plate. A range of concentrations of CCL19/CCL21 (0–200 ng/ml, Propetech) was added to the medium, and the cells were cultured for 48 h. Mesangial (M) cells, which display a proliferative response upon exposure to CCL19 [27], were kindly provided by Professor Mei Changlin (Shanghai Eastern Hepatobiliary Hospital) as a positive control. Then, 100 μl of fresh medium in 10 μl of the CCK-8 solution was added to each well and incubated at 37°C for 2 h. The absorbance (A) at 490 nm was measured. All assays were performed in quadruplicate, and three individual experiments were performed. The data are expressed as the mean values ± SD of 4 wells per treatment.

### Assessment of fibroblast mineralization

1 × 10^6^ fibroblasts from one AS LT were seeded on 6-well plates. Five groups were assigned: osteogenic differentiation medium as a positive control, CCL19 (10 ng/ml, according to previous studies [16] and the manufacturer’s recommendations), CCL21 (10 ng/ml), CCL19/CCL21 (both 10 ng/ml), and PBS as a negative control (Grow group). After culturing for 48 h, total RNA was extracted to determine the mRNA expression levels of the osteogenesis-specific transcription factors Runx-2 and Osterix and the osteogenic differentiation markers IBSP, COL1, ALP and OCN. On days 2, 4 and 6, the culture supernatants were collected to detect of the levels of ALP and OCN (N-MID ELISA kit, Immuno-diagnostic Systems Inc, Fountain Hills, AZ, USA) via ELISA. All measurements were performed in duplicate.

### Statistical analysis

All data are expressed as the means ± standard deviation (SD). For statistical comparisons of two groups of samples, the Mann–Whitney U-test was used. Alternatively, comparisons of continuous variables were performed using Student’s two-tailed t-test using SPSS 17.0 software; p < 0.05 was considered to be significant. The relative gene expression levels were determined according to the 2^-ΔΔ^C_t_ method, and the results were expressed as the fold-change compared to the negative control.

## Results

### Higher expression of CCL19 and CCL21 in AS LT than in OA LT

**Figure 1:**
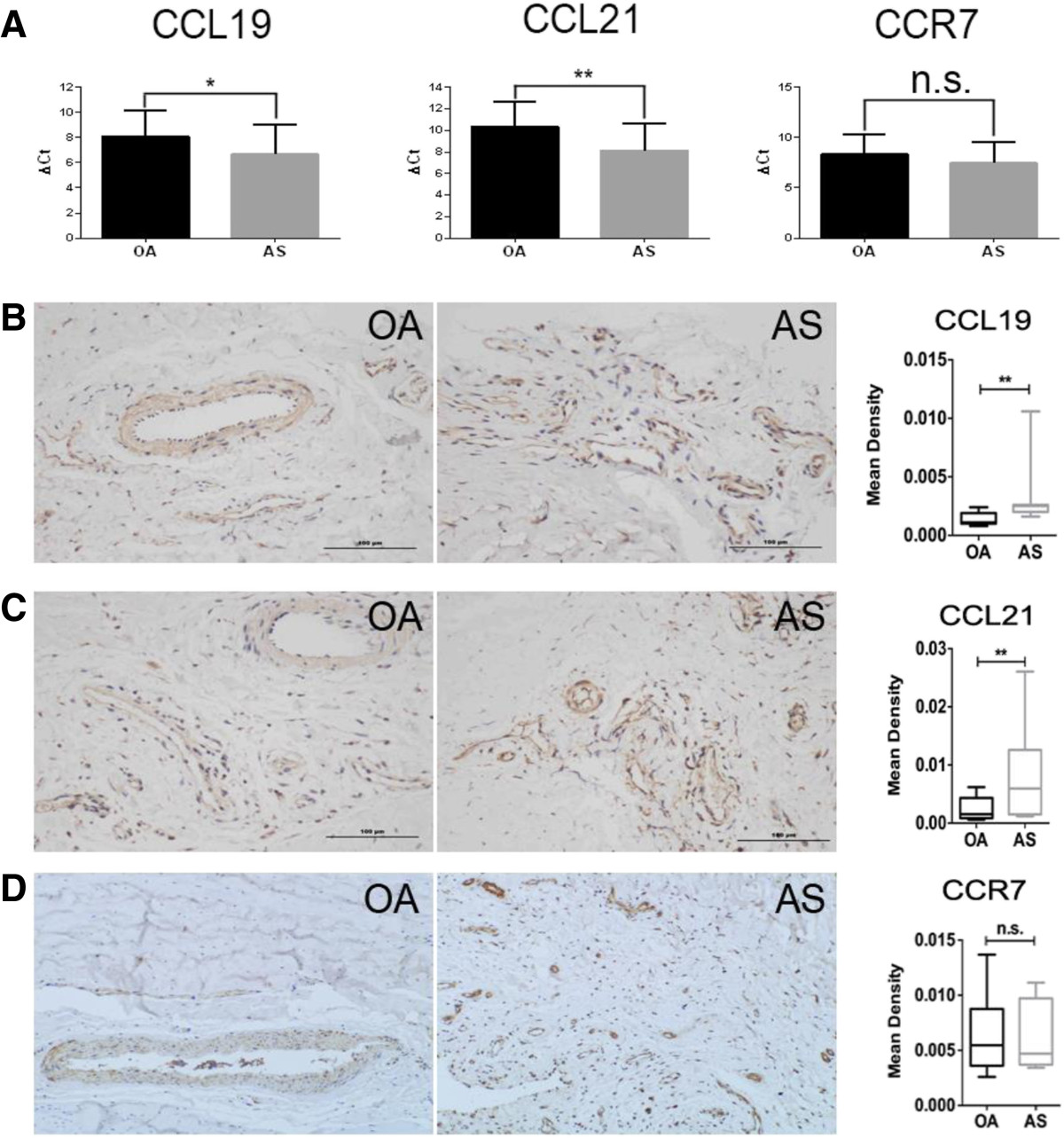
**Expression of CCL19, CCL21 and CCR7 in AS and OA ligament tissue (LT). (A)** RT-PCR analysis revealed the mRNA expression levels of CCL19, CCL21 and CCR7 in AS (n = 31) LT compared to OA (n = 19) LT (P = 0.034, 0.004 and 0.155, respectively). The data are expressed as the ΔCt value (Ct target gene-Ctβactin ) between the OA and AS groups, and the Mann–Whitney U-test was performed. **(B-D)** AS (n = 15) and OA (n = 15) LT were stained with rabbit anti-human CCL19 **(B)**, rabbit anti-human CCL21 **(C)** or rabbit anti-human CCR7 **(D)**, and the immunostaining was expressed as the mean density ± standard deviation (SD). The expression levels of CCL19, CCL21 and CCR7 were compared between the AS and OA groups, with P values of 0.016, 0.023 and 0.191, respectively. *P < 0.05; **P < 0.01; n.s. = no significance. The original magnification is × 200 in B, C, and D.

We first investigated the expression of CCL19/CCL21 in AS and OA hip LT via real-time PCR and IHC. RT-PCR analysis revealed significantly increased transcriptional expression levels of CCL19/CCL21 (Figure 1A) in AS LT compared to OA LT (P = 0.034 and P = 0.004). The smaller the ΔCt value, the higher the mRNA expression level. The IHC results further validated the higher expression of these two chemokines in the AS group than the OA group (Figure 1B-C, P = 0.016 and P = 0.023). However, the expression their receptor CCR7 did not display a significant difference between the two groups (Figure 1A and D, P = 0.155 and P = 0.191). Interestingly, we found that CCL19/CCL21 were primarily expressed on cells surrounding the capillaries, which likely corresponds to vascular endothelial cells, indicating a more important role in angiogenesis and migration of cells such as macrophages and dendritic cells. Previous studies found a proangiogenic effect of CCL19/CCL21 via the induction of secretion of vascular endothelial growth factor (VEGF) and angiotensin I (Ang-I) in fibroblasts [16]. Combined with our results, we propose that the expression of CCL19/CCL21 in AS LT is involved in both inflammatory cell migration and abnormal angiogenesis.

### CCL19 and CCL21 does not exert a significant proliferative effect on ligament fibroblasts

**Figure 2:**
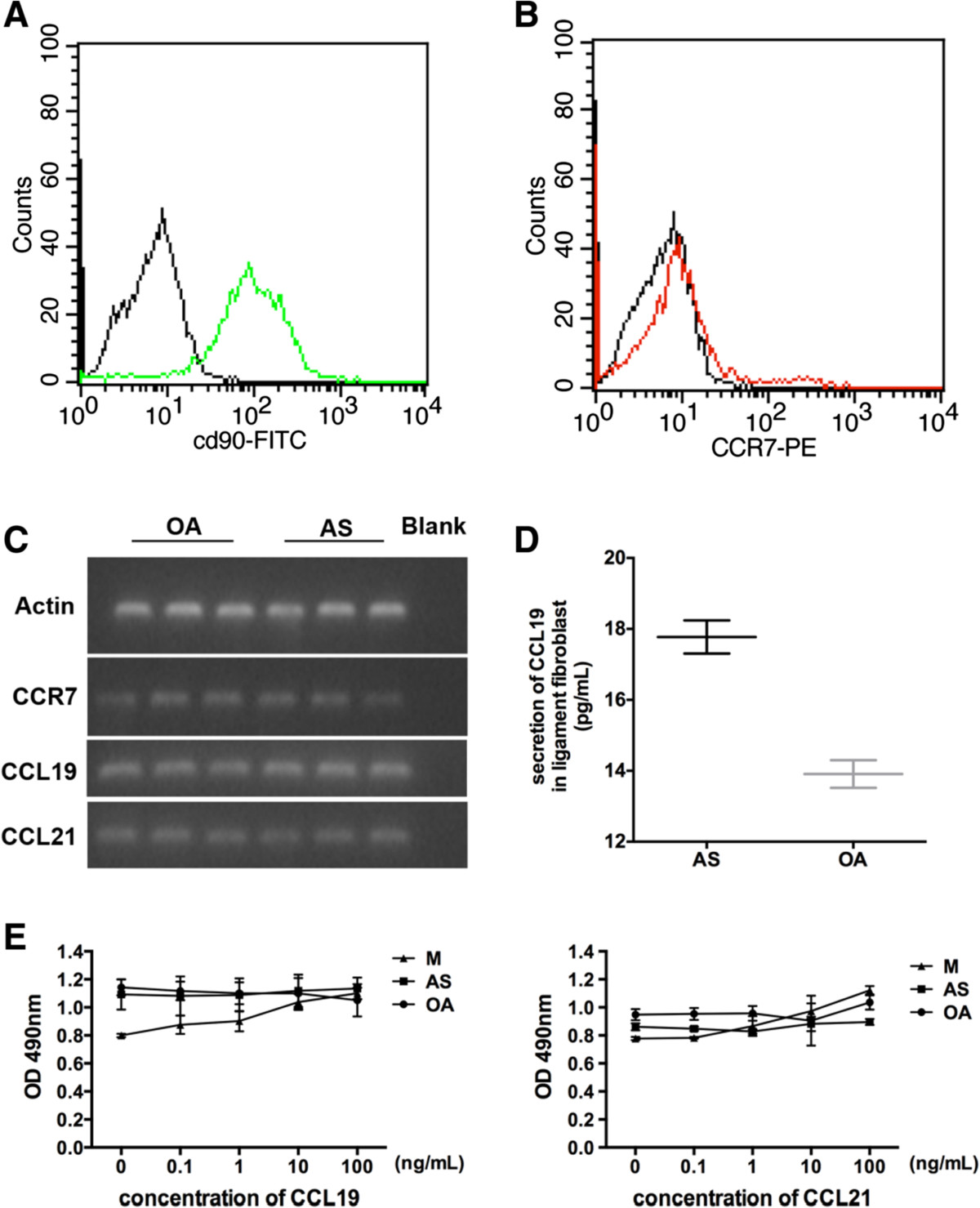
**Expression levels of CCL19, CCL21 and CCR7 in fibroblasts and their role in fibroblast proliferation. (A)** Morphology of in vitro cultured fibroblasts via light microscopy. The original magnification is × 100. **(B)** A representative image of the expression of the fibroblast marker CD90 and CCR7 in AS LT fibroblasts. **(C)** RT-PCR and nucleic acid gel electrophoresis analyses revealed the expression of CCL19, CCL21 and CCR7 in 3 AS and 3 OA ligament fibroblast cultures. Blank corresponds to a control well containing no template cDNA. **(D)** ELISA revealed the secretion of CCL19 in 3 AS and 3 OA fibroblast cultures, and the experiments were repeated three times in duplicate. The data were calculated as the average value (CCL19 concentration) ± Standard deviation (SD). **(E)** Incubation of fibroblasts in various concentrations of CCL19 and CCL21 for 48 h. Cell proliferation was analyzed via the CCK8 assay as described in the Methods section. Cells cultured under standard growth conditions (growth medium) were used as the negative control. Additionally, mesangial (M) cells were used as the positive control. Changes in the proliferative activity were expressed as the values relative to the negative controls. Each result represents the mean ± SD of 4 parallel incubations for each condition. Comparable results were obtained from three independent experiments.

We next examined the expression of their receptor CCR7 on the ubiquitous LT fibroblasts. The fibroblasts in passages 3 to 8 were used to perform the expression and functional analyses. The fibroblasts from the AS and OA LT did not display any differences in morphology based on light microscopy (Figure 2A). FACS analysis revealed strong expression of the fibroblast marker CD90 but relatively weak expression of CCR7 (Figure 2B). However, RT-PCR and nucleic acid gel electrophoresis clearly confirmed the expression of CCL19, CCL21 and CCR7 in LT fibroblasts from AS (n = 3) and OA patients (n = 3) (Figure 2C). These results indicated that increased levels of CCL19/CCL21 could stimulate LT fibroblast via CCR7 signaling. Moreover, we also detected secretion of CCL19 in the supernatants of 3 AS fibroblast cultures (Figure 2D), but CCL21 was not detected. This result implies that CCL19/CCL21 might be differently regulated in AS LT fibroblasts.

Because the abnormal proliferation of fibroblast-like cells was reported in ankle/tarsal joints of ankylosing enthesitis male mice [28] and CCL19/CCL21 induce proliferative effects on various cell types [15], we examined whether stimulation with CCL19/CCL21 for 48 h affected fibroblast proliferation in LT. As shown in Figure 2E, at various concentrations of CCL19/CCL21, the A490 value did not significantly differ, and only a limited proliferative effect was detected in both AS and OA LT fibroblasts (P > 0.05, each compared to the Grow group). This result suggests that neither CCL19 nor CCL21 affected fibroblast growth.

### Promotion of osteogenesis in CCL19 and CCL21-stimulated AS fibroblasts

**Figure 3:**
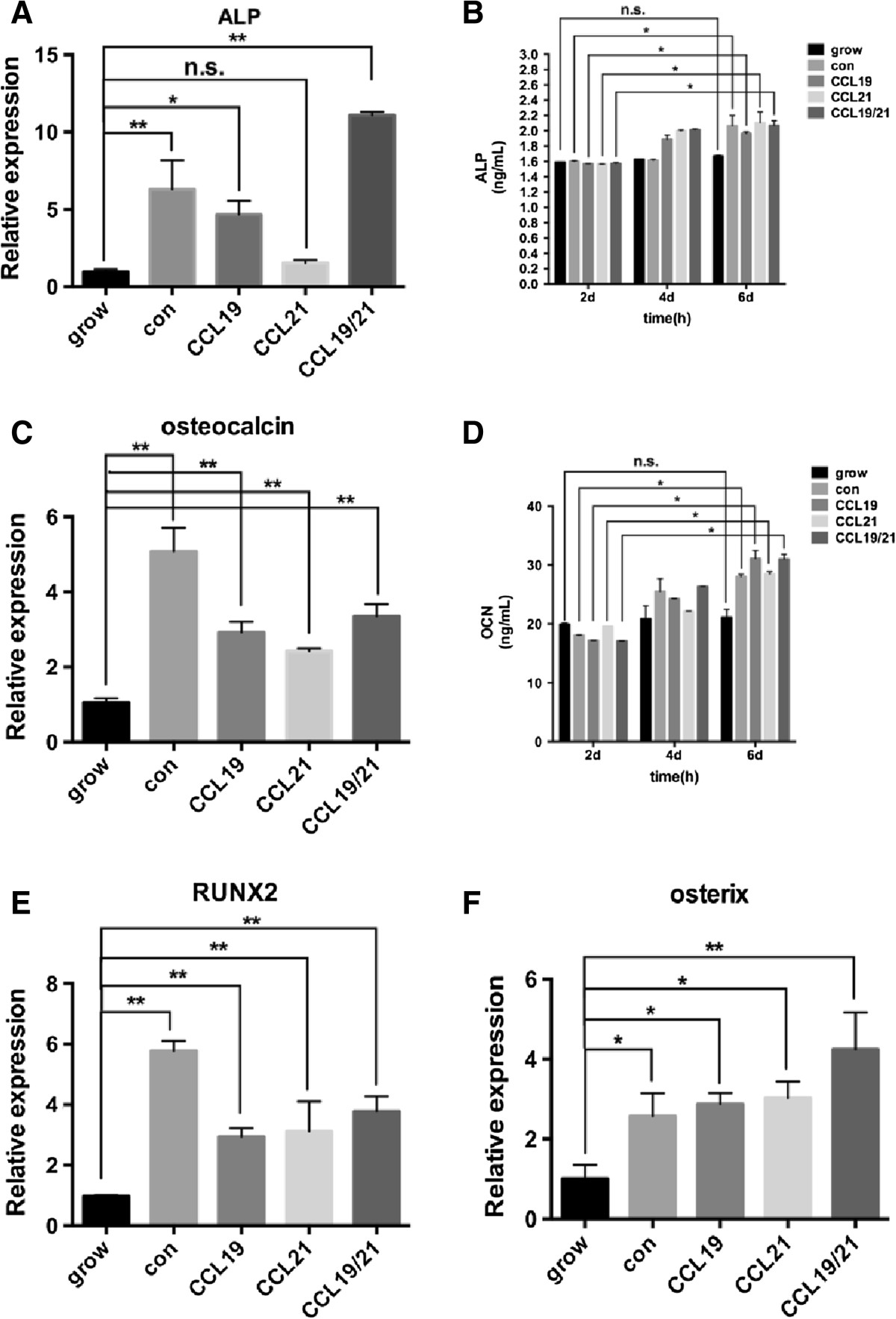
**Effect of exogenous CCL19/CCL21 treatment on the osteogenic potential of AS LT fibroblasts after stimulation for various periods.** Five groups were assigned: Grow (standard culture medium), control (osteogenesis-induced culture medium), CCL19 (standard culture medium supplemented with 10 ng/ml CCL19), CCL21 (standard culture medium supplemented with 10 ng/ml CCL21) and CCL19/21 (standard culture medium supplemented with 10 ng/ml CCL19 and 10 ng/ml CCL21). ALL data are compared to the Grow group, and the unpaired t-test was performed. **(A)** Changes in the mRNA levels of the early bone marker ALP in AS fibroblasts after stimulation for 48 h. The CCL19/CCL21 group displayed the maximal effect (P < 0.01), but the expression of CCL21 did not display a significant change. **(B and D)** ELISA revealed the secretion of ALP and OCN at various time points (2, 4 or 6 days). **(C)** Changes in the OCN mRNA levels after CCL19/CCL21 stimulation for 48 h. **(E-F)** The mRNA levels of the osteogenesis regulators Runx2 and osterix were also up-regulated by CCL19 or CCL21 treatment for 48 h. *P < 0.05; **P < 0.01; n.s. = no significance.

Inflammation and ligament ossification are common symptoms in AS patients. Here, we discovered the pro-osteogenic potential of CCL19/CCL21 in AS LT fibroblasts. Five groups were assigned: Grow, control, CCL19, CCL21 and CCL19/CCL21. The concentration of each chemokine was 10 ng/ml. After treatment for 48 h, ALP mRNA expression was up-regulated more than 2-fold in the control, CCL19 and CCL19/CCL21 groups (Figure 3A, P = 0.008, P = 0.026 and P = 0.0042, respectively) compared to the Grow group, but the CCL21 group did not display a significant difference compared to the Grow group (P = 0.115). The ELISA results revealed increased secretion of ALP (Figure 3B) over the three time points (2, 4 and 6 days). However, two other bone markers, COL1 and IBSP, did not display this change (data not shown) in response to these two chemokines. The expression of the interim bone marker OCN demonstrated changes in both the mRNA and protein levels that were similar to those of ALP (Figure 3C-D). In addition, the expression of the key regulators Runx-2 and osterix, which modulate osteoblast differentiation, were also significantly up-regulated after CCL19/21 stimulation compared to the Grow group (Figure 3E-F). Because ALP and OCN are regulated by Runx-2 and osterix, these findings suggest that CCL19/CCL21 promote the ossification of AS ligament fibroblasts via Runx-2 and osterix.

### Clinical significance of CCL19 and CCL21

**Figure 4:**
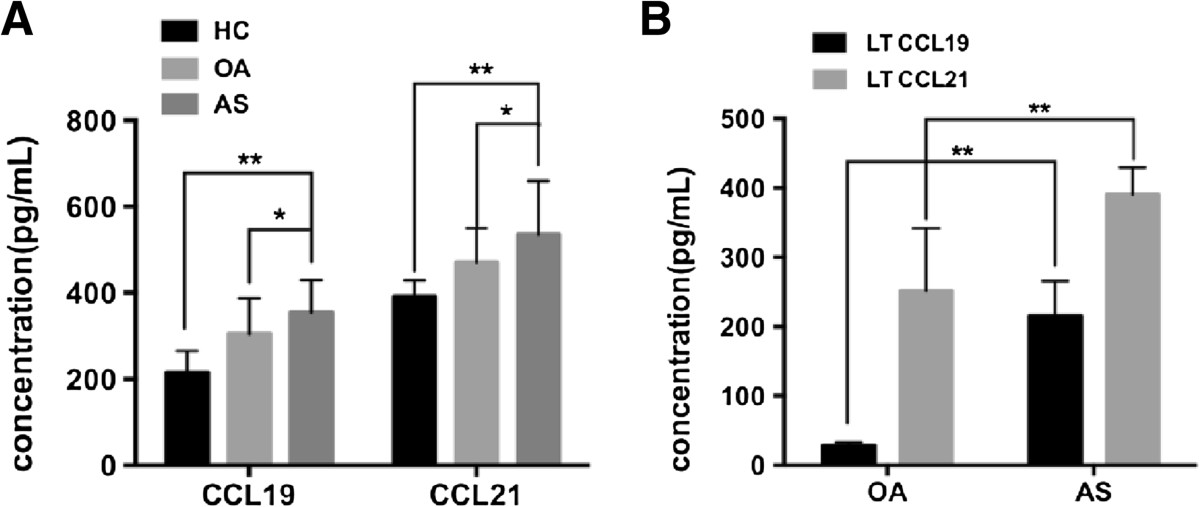
**CCL19 and CCL21 levels in serum and ligament tissue (LT). (A)** Serum CCL19/CCL21 levels in AS (n = 42), OA (n = 29), and healthy controls (HC, n = 16). The CCL19 level in the AS group was clearly higher than that in the OA and HC groups (AS *vs* OA, P = 0.011 and AS *vs* HC, P = 0.003). The CCL21 level displayed a similar distribution (AS *vs* OA, P = 0.039 and AS *vs* HC, P = 0.0075). **(B)** The levels of CCL19/CCL21 in AS (n = 27) and OA (n = 12) ligament tissue. The CCL19 level was significantly increased in AS ligament tissue compared to OA LT (AS *vs* OA, P = 0.024), and CCL21 level displayed a similar difference (AS *vs* OA, P = 0.041).*P < 0.05; **P < 0.01.

The emerging role of CCL19 or CCL21 in the clinic has been identified for many diseases. Thus, we explored the possible relationship between CCL19/CCL21 and inflammation (based on ESR, CRP and the VAS score), function (BASFI), disease activity (BASDAI) and global assessment (BAS-G, disease duration, morning stiffness) in AS patients. The serum CCL19/CCL21 levels were significantly higher in AS patients than in OA patients and healthy controls (HC) and were higher in OA patients than in HC (Figure 4A, both P < 0.05 compared to the OA or HC group). The CCL19/CCL21 levels were both higher in the AS LT compared to the OA LT (Figure 4B, each chemokine compared to the OA group, both P < 0.01). Correlation analysis revealed that the serum CCL19 level was closely associated with the serum CCL21 level (r = 0.546, P < 0.01) and was also correlated with the VAS score (r = 0.327, P = 0.035) and the disease duration (r = 0.316, P = 0.041) (Table 3) in AS patients. No correlation was detected between the LT CCL19/CCL21 levels and any clinical indicator (data not shown). Furthermore, the serum CCL19/CCL21 levels were not correlated to the LT CCL19 or CCL21 levels in AS patients, which displayed a weak relationship between the different sites. These results reveal that the increased serum CCL19/CCL21 levels are closely correlated and that CCL19 may be more influential in predicting global clinical manifestations in AS patients.

**Table 3:** **CCL19 and CCL21 levels in AS (n = 42) serum and correlation to clinical characteristics**

	Serum CCL19		Serum CCL21	
	r	P value	r	P value
Serum CCL19 level	NA	NA	0.546	**0.003**
Serum CCL21 level	0.546	**0.003**	NA	NA
Age	0.167	0.291	0.152	0.337
ESR	−0.036	0.820	0.062	0.698
CRP	0.191	0.226	0.226	0.150
BASDAI	0.233	0.137	0.182	0.250
BASFI	0.245	0.118	0.185	0.241
BASG	0.234	0.136	0.284	0.069
VAS	0.327	**0.035**	0.181	0.252
Staff time	0.102	0.520	0.251	0.109
Disease duration	0.316	**0.041**	0.254	0.105

Abbreviations are the same as those referred to above. Bold values means P < 0.05.

## Discussion

In this study, we detected increased expression of CCL19/CCL21 in AS hip LT. IHC staining of CCL19/CCL21, as well as their distribution, indicate that vascular endothelial cells and fibroblasts may represent the primary source of CCL19/CCL21. However, in vitro cultured AS ligament fibroblasts secreted only a small amount of CCL19 and no CCL21. This indicates that other cells, such as vascular endothelial cells, are likely the source of CCL19/CCL21. Moreover, inflammatory macrophages in enthesis and inflammatory factors, such as TNF-α, LPS, which regulate the expression of CCL19/CCL21 in synovial fibroblasts [16], may also similarly mediate the production of CCL19/CCL21. However, additional evidence is required.

Because fibroblasts are the principal cell type in LT and are closely associated with ligament ossification in AS patients [22], we examined whether CCL19/CCL21 exert an effect on LT fibroblast proliferation or ossification, both of which are reported in AS. Previous studies have demonstrated the various proliferation effects of CCL19/CCL21 on human M cells, CD4 and CD8 T cells, bone marrow and cord blood CD34+ cells [15, 27, 29–31]. A recent study reported a minor role of CCL19/CCL21 on the survival, proliferation or migration of adult neural precursor cells [32]. Alternatively, fibroblasts from various organs displayed enhanced or inhibited proliferation in response to stimulation, such as hypoxia, Beta2-adrenergic receptor agonist treatment and EGF treatment [4, 33, 34]. Here, we detected no apparent proliferative effect of CCL19/CCL21 on ligament fibroblasts in the 0–200 ng/ml concentration range. The reason for this result may be that ligament fibroblasts are terminally differentiated cells with limited proliferation capability.

Syndesmophytes are evident in AS and, ligament fibroblasts are involved in their formation [22]. Many investigators have identified the ossification tendency of fibroblasts in LT. Cytokines such as CCL3 has been found to inhibit osteogenesis in myeloma [35]. In this study, we found higher expression of bone markers, such as ALP and OCN, and the transcription factors Runx-2 and osterix after stimulation of AS fibroblasts with CCL19/CCL21. Runx-2 and osterix are key regulators of osteoblast maturation and differentiation and modulate the expression of bone markers, including ALP, COL1, IBSP and OCN [36]. Our results provide an innovative role of CCL19/CCL21 in fibroblast ossification, thus presenting new research targets for ligament lesions in AS. However, the precise mechanism by which CCL19/CCL21 affect mineralization via Runx-2 or osterix is not certain. In RA synovial fibroblasts, CCL19 expression is primarily modulated by lipopolysaccharide (LPS), tumor necrosis factor-α (TNF-α), interleukin-1 beta (IL-1β) and IL-8, whereas CCL21 is modulated by TNF-α. Exogenous CCL19/CCL21 treatment up-regulated the expression of VEGF and Ang-I, both of which are crucial mediators of angiogenesis [16]. Parallel studies of RA and OA fibroblasts have also found that CCL19/CCL21 mediated migration [37]. In our study, we detected significantly increased VEGF mRNA expression following CCL19/CCL21 stimulation (data not shown). Ossification and angiogenesis are closely correlated, and VEGF is an essential mediator of both of these processes [38]. Furthermore, VEGF is also regulated by osterix [39]. Based on the above findings, we speculate that inflammatory factors, such as LPS or TNF-α, may be regulate CCL19/CCL21, and CCL19/CCL21 promote fibroblast ossification via the Runx-2-osterix-VEGF pathway. However, further evidence is required to validate this hypothesis, and additional studies are necessary.

In this study, we detected higher levels of serum CCL19/CCL21 in both AS and OA patients compared to HC. CCL19/CCL21 were reported to predict the inflammatory state in some diseases [19, 20], but we did not detect any correlation between CCL19/CCL21 expression and inflammation or functional characteristics in AS patients. The serum CCL19/CCL21 levels are closely associated, which might indicate a combined effect of pathogenicity. Furthermore, the CCL19 levels correlated to two disease-related global and activity indicators: the VAS score and the disease duration. The VAS score reflects the pain level, and the disease duration may reflect the onset of disease. This result indicates that CCL19 is a relative indicator of early onset in AS patients.

There are some limitations to our study. First, pathological studies demonstrated that multiple cell types, including fibroblasts, periosteal cells, endothelial cells and stem cells, are present in LT, so other cells that express CCR7 should also be considered. Second, all of the patients in our study were admitted for joint replacement surgery and exhibited severely restricted hip activity and a poor BASFI score (average BASFI = 62.24, indicating severe functional limitations), and these patients do not represent the entire AS population. Third, there is a significant difference in the average age between the AS and OA groups, which may influence the results. Fourth, the precise concentrations of CCL19/CCL21 in AS LT (n = 10) and peripheral blood are similar to those of RA synovium and peripheral blood [16], but ossification is not observed in RA patients. Two reasons may account for these results: ossification is caused by changes in the gene expression level in AS, and CCL19/CCL21 may accelerate this progress; and the concentration of CCL19/CCL21 used in this study, 10 ng/ml, which is far more than the levels in LT, induced the activation of ossification in cultured fibroblasts in vitro. In addition, the number of AS patients included in our study is small for correlation analysis. Further investigation using larger sample sizes would provide more convincing results.

In summary, we observed higher expression of CCL19/CCL21 in AS LT. The increased CCL19/CCL21 displayed pro-ossification potential but did not affect the proliferation of AS ligament fibroblasts. Moreover, the serum CCL19 levels significantly correlated to the serum CCL21 levels, the VAS score and the disease duration, which reveals its important role in global disease assessment of AS patients.

## Conclusion

CCL19/CCL21, two chemokine that were higher expressed in AS LT and showed a pro-ossification potential, were also elevated and closed associated in serum samples of AS patients.

## Author’s information

Common communication: Miao Min Yong.

## Authors’ original submitted files for images

Below are the links to the authors’ original submitted files for images. Authors’ original file for figure 1 Authors’ original file for figure 2 Authors’ original file for figure 3 Authors’ original file for figure 4

## Abbreviations

CCL19: Chemokine (C-C motif) ligand 19
CCL21: Chemokine (C-C motif) ligand 21
AS: Ankylosing spondylitis
LT: Ligament tissue
CCR7: Chemokine (C-C motif) receptor 7
OA: Osteoarthritis
ALP: Alkaline phosphatase
OCN: Osteocalcin
COL1: Collagenase I
IBSP: Integrin-binding sialoprotein
Runx-2: Runt-related transcription factor-2
HO: Heterotopic ossification
RA: Rheumatoid arthritis
ESR: Erythrocyte sedimentation rate
CRP: C-reactive protein
BASDAI: Bath Ankylosing Spondylitis Disease Activity Index
BASFI: Bath Ankylosing Spondylitis Functional Index
VAS: Visual analog scale
BAS-G: Bath Ankylosing Spondylitis Patient Global Score.
