# Supplementary figures and images for: Increased CCL19 and CCL21 levels promote fibroblast ossification in ankylosing spondylitis hip ligament tissue
Source: BMC Musculoskelet Disord. 2014 Sep 26;15:316. doi: 10.1186/1471-2474-15-316 (PMC4190335; doi:10.1186/1471-2474-15-316)

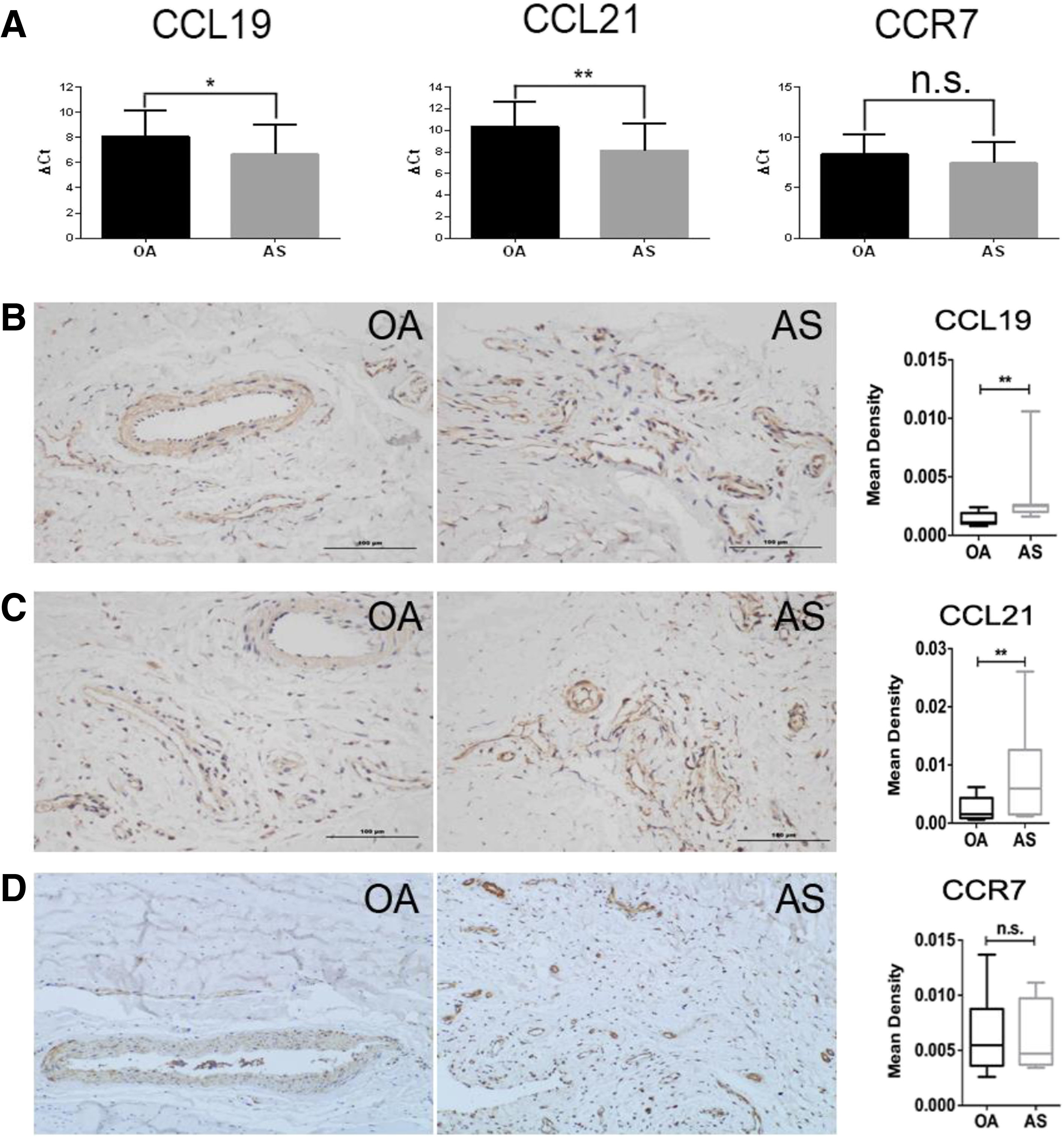

Supplement: Supplementary file 1 — Authors’ original file for figure 1 [file 12891_2014_2256_MOESM1_ESM.tif]

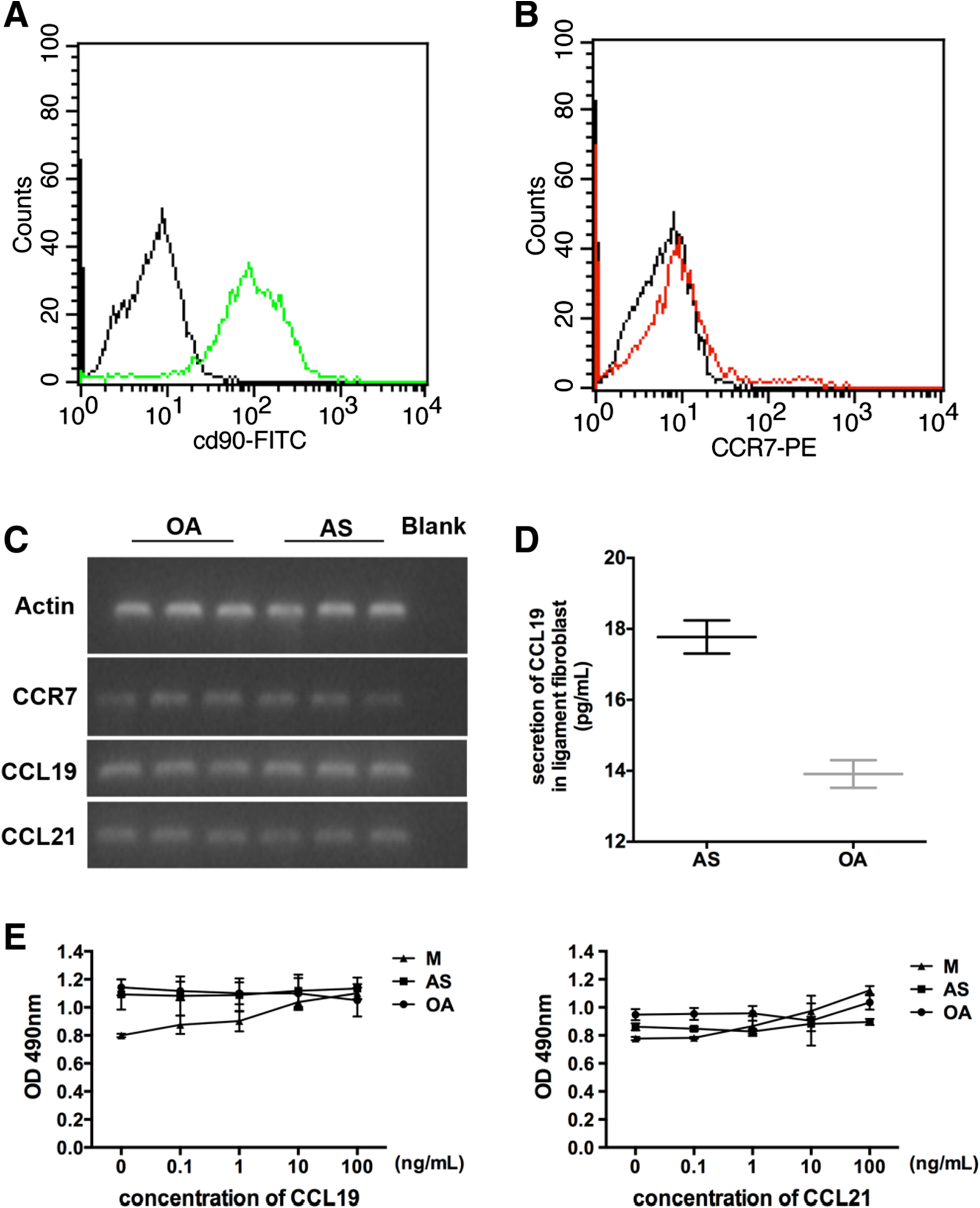

Supplement: Supplementary file 2 — Authors’ original file for figure 2 [file 12891_2014_2256_MOESM2_ESM.tif]

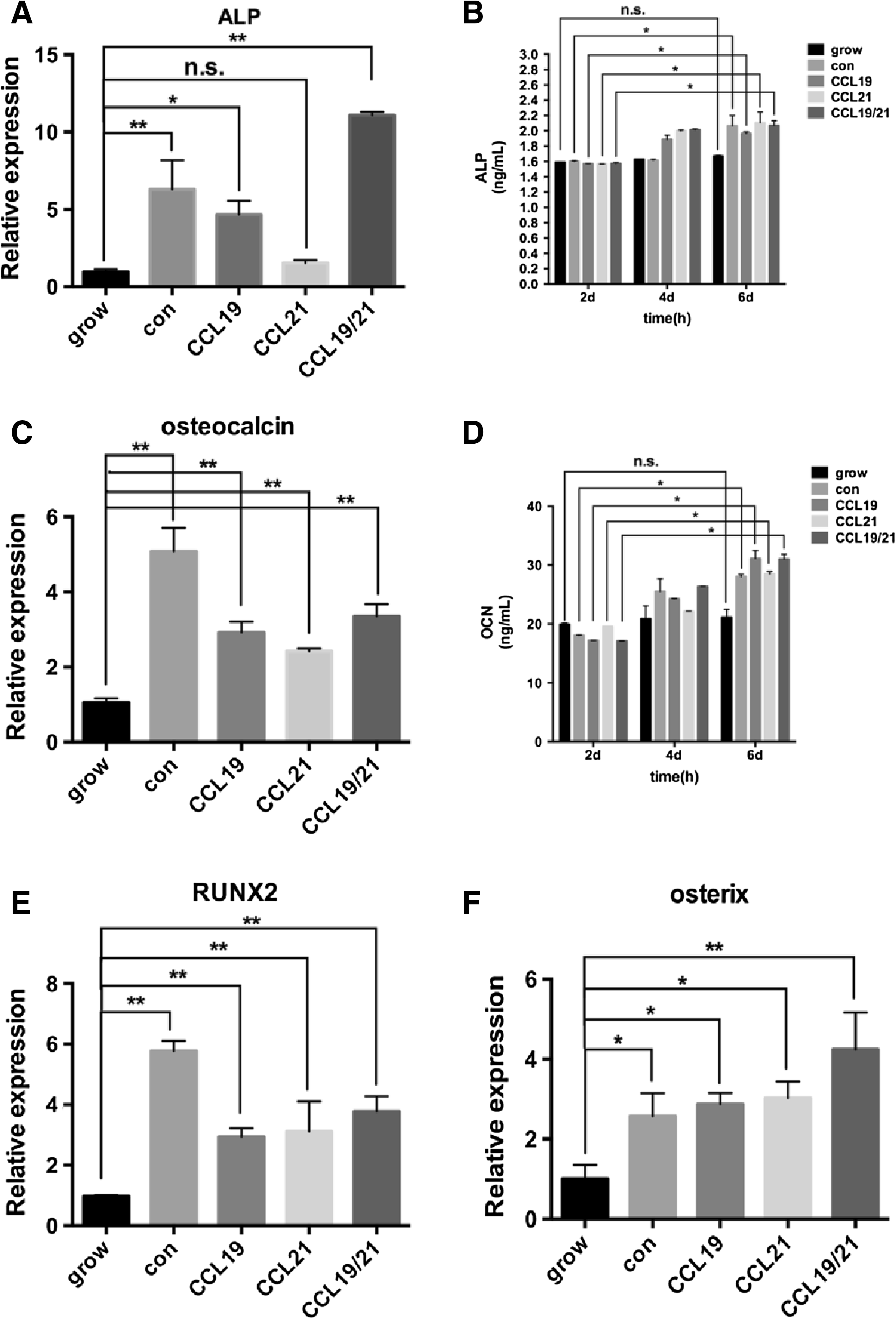

Supplement: Supplementary file 3 — Authors’ original file for figure 3 [file 12891_2014_2256_MOESM3_ESM.tif]

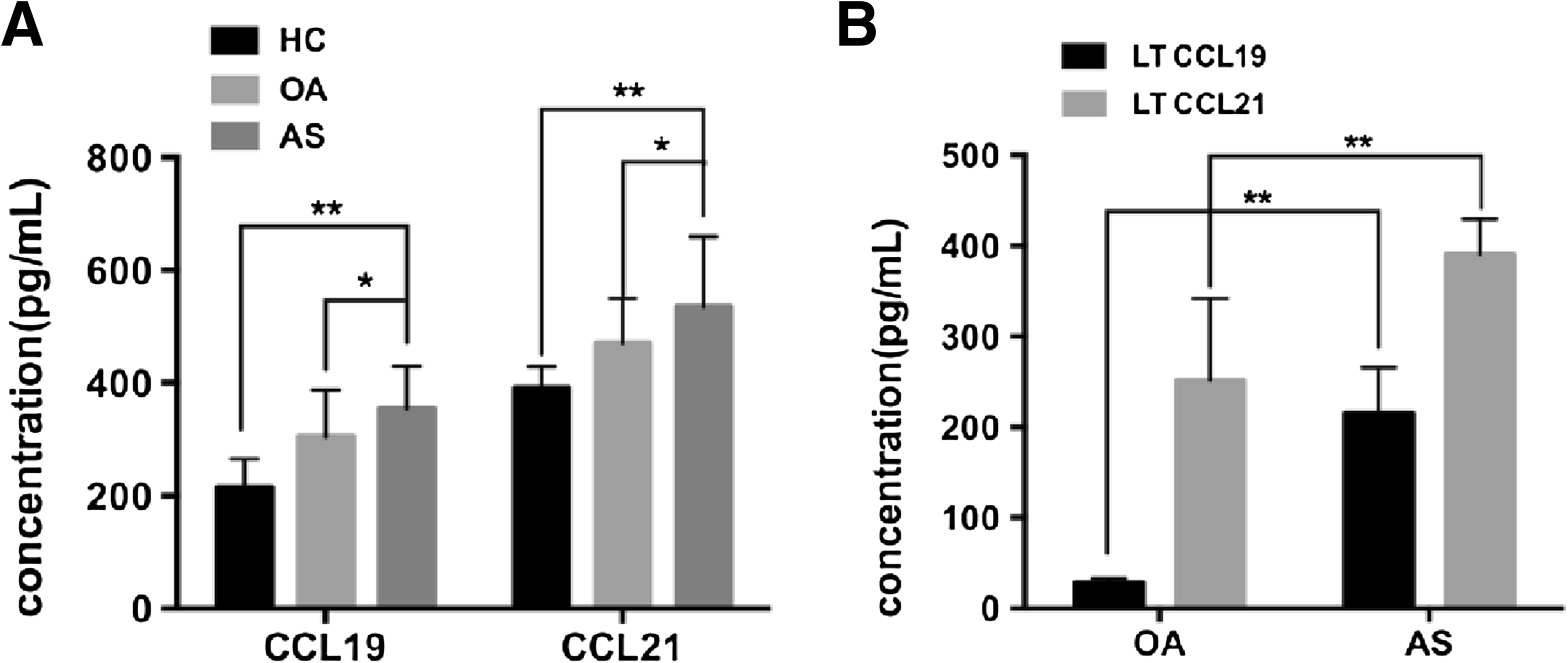

Supplement: Supplementary file 4 — Authors’ original file for figure 4 [file 12891_2014_2256_MOESM4_ESM.tif]
